# Effect of layering characteristics on compressive mechanical properties and damage mechanisms of rocks: Experiments and models

**DOI:** 10.1371/journal.pone.0318603

**Published:** 2025-02-21

**Authors:** Chong Chen, Aixiang Wu, Shaoyong Wang, Wutian Gong, Wei Sun, Tong Gao

**Affiliations:** 1 Key Laboratory of the Ministry of Education of China for High-efficient Mining and Safety of Metal Mines, University of Science and Technology Beijing, Beijing, China; 2 School of Resources and Safety Engineering, University of Science and Technology Beijing, Beijing, China; 3 Faculty of Land and Resources Engineering, Kunming University of Science and Technology, Kunming, China; Guizhou University, CHINA

## Abstract

The investigation of layered rock mechanical properties is important for rock stability analysis. To examine the effects of layer inclination angle (LIA) and layer thickness (LT) on the compressive mechanical properties and damage mechanism of layered rock, this paper proposes a new method of preparing layered rock specimens using similar materials and conducting uniaxial compression tests. At the same time, PFC2D numerical analysis software is used to establish the discrete element numerical model of layered rock under uniaxial compression to deepen its microscopic damage mechanism. The results show that the LIA significantly affects the anisotropic mechanical properties of the rock, and the different LIA lead to significant differences in the crack extension paths and failure modes, which can be summarized into four types of cracks and four failure modes. The increase in LT under the effect of different LIA shows different mechanisms of action, which is enhanced in 0° and 90°, weakened in 45° and 75°, and insignificant in 15°, 30° and 60°. In addition, the expansion of micro-cracks in layered rocks are all dominated by interlayer shear cracks first, and then conduct to the rock portion to tensile cracks. The findings of this research offer valuable insights for stability assessment and design of layered rock structures in engineering applications.

## 1. Introduction

Layered rock masses are widely distributed in various continents and marine environments around the world, including shale, gneiss, schist, slate, phyllite, and coal [1–4]. It is a rock structure composed of multiple parallel or nearly parallel rock layers, usually exhibiting significant anisotropy, which has attracted much attention in the fields of engineering and geology, especially in rock deformation, collapse, landslide, subsidence, etc., [5–9].

Specimen preparation is the primary challenge in studying the impact of layered structure on the mechanical behavior of rock. Researchers find it difficult to obtain a sufficient number of intact specimens from natural layered rock. Therefore, rock like materials have become a necessary alternative for studying layered rocks, which exhibit stress-strain evolution processes similar to natural rocks in mechanical tests [10–12]. The most commonly used rock-like materials are cement, sand and gypsum [13,14]. The specimens are prepared by casting the rock-like materials in layers and then drilling holes for samples at different angles [15]. Although this method is convenient to implement, but the bonding strength between the layers is excessively high, and the specimen preparation process requires further refinement.

By combining rock-like materials with natural layered rocks, researchers have conducted extensive studies on mechanical behavior. SI et al. [16] indicates that as the LIA increases from 0° to 90°, the failure mode gradually shifts from tensile splitting along the bedding planes to shear slipping along the weaker bedding planes. Ma et al. [17] predicted that the fracture compression caused by anisotropic tensile strength decreases nearly 10%, and as the degree of anisotropy increases, the fracture compression decreases further. Ramamurthy [18] classified the anisotropy of rocks into “U” type, “undulatory” type, and “shoulder” type, respectively, summarized the failure mode as following: tensile splitting across the rock matrix, shear slip along the interlayer and mixing of the two. At the same time, various failure criteria were proposed to describe the transverse strength anisotropy. Yang et al. [19] found that the apparent friction angle and cohesion show dispersion with respect to the LIA. Aminpure et al. [20] investigated the effects of different roughness levels and orientation angles of discontinuities on the parameters of jointed rock failure criteria. Tiwari et al. [21] examined the slope of post-peak stress-strain curves in rocks with one set of non-persistent joints and two sets of persistent joints. Wang et al. [22] found that bedding strength significantly influences tensile strength and failure models, whereas the spacing of the layers has a limited impact. Layered rock also show various anisotropies in tensile or flexural mechanical properties [23]. Shen et al. [24] summarized twelve crack types, eight failure modes and five axial stress-strain curve types based on physical tests and existing research. These findings have identified the impact of the layered structure on the mechanical behavior of rock, but they are still not comprehensive enough.

In recent years, PFC has been widely utilized for constructing models of rock masses with diverse layer properties by using a discrete element method to simulate material response, which effectively replicating the mechanical behavior of materials based on interaction forces between particles. This numerical method provides a discrete description of the continuous motion of material particles, allowing the PFC uniaxial compression code to accurately simulate material stresses and deformations. Yang et al. [25] and Cheng et al. [26] successfully simulated the compression damage behavior and failure modes of layered rock using PFC, yielding results closely matching physical test. Therefore, PFC modeling has been demonstrated to be a promising method for gaining a better understanding of the mechanical behavior mechanisms of layered rock [27–30]. The combination of physical experiments and numerical modeling is the most common and effective approach for studying rock mechanics [31–35]. Other test methods, such as DIC and CT, have been used to study the crack development patterns of layered rock during failure [36–38].

This paper focuses on the impact of LIA and LT of the layered structure on the mechanical properties and damage mechanisms of rock. A novel method for preparing layered rock specimens is proposed, and uniaxial compression tests and PFC numerical simulations were conducted on specimens. The effects of LIA and LT on UCS and failure modes were evaluated. The failure models were summarized, and the failure mechanisms were analyzed through macro and micro cracks. The study results offer guidance for assessing stability and developing support strategies in engineering projects involving layered rock.

## 2. Materials and methods

### 2.1. Similar materials

**Fig 1:**
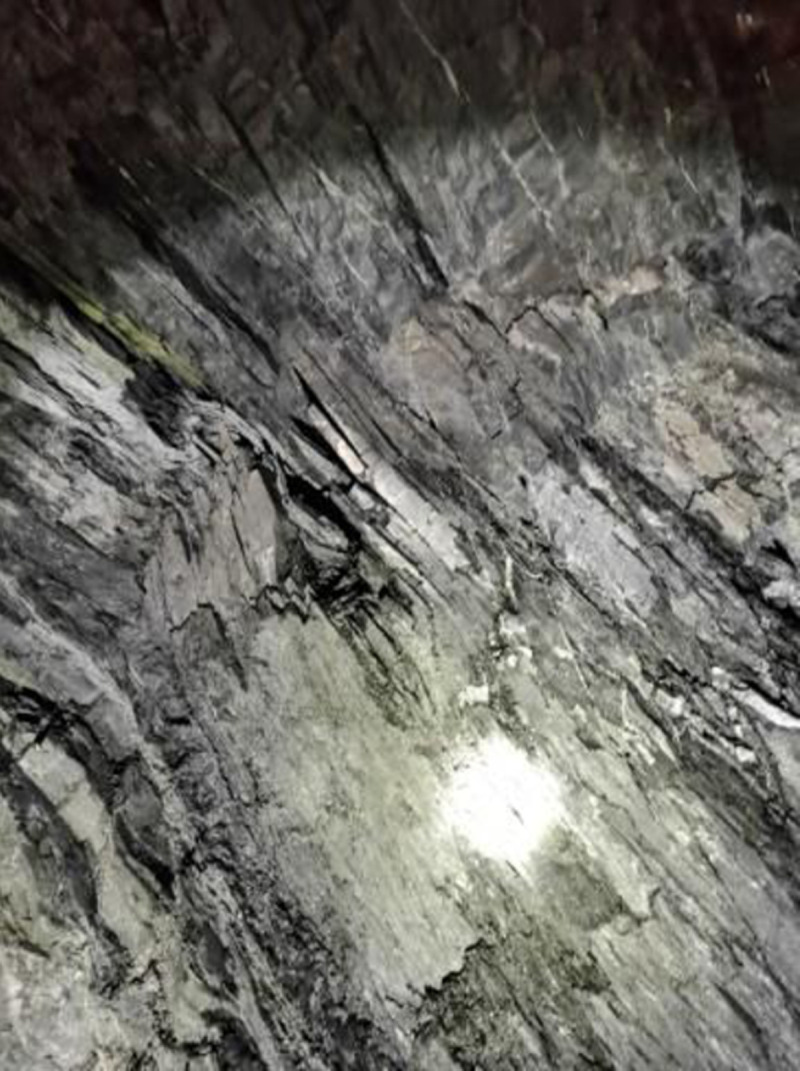
Layered phyllite.

The rock simulated in this experiment was obtained from the Yongchang Lead-Zinc Mine in Yunnan Province, China, as shown in Fig 1. The UCS of rock is 25.03 MPa, and the LT ranges from 1 to 12 cm.

**Fig 2:**
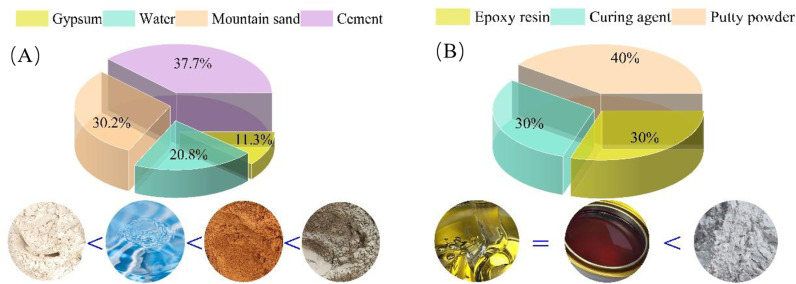
Material proportion. (A) Rock-like material, (B) Interlayer bonding material.

In this simulation experiment, the layered rock specimen was divided into two parts: the rock portion and the interlayer joint surfaces. The rock portion was modelled using a mixture of mountain sand, P.O42.5 ordinary portland cement, gypsum, and water. Among them, mountain sand was used as the coarse aggregate, and gypsum and cement were used as the binder. The material proportion for making layered rock specimen is 1:0.8:0.3:0.55 (cement: sand: gypsum: water), as illustrated in Fig 2A. The UCS of the specimen is consistent with the prototype, measuring 25 ±  0.6 MPa.

The interlayer bonding material was prepared by mixing epoxy resin, a curing agent, and putty powder. Epoxy resin served as the primary cementing component due to its high bonding strength and short setting time. The addition of putty powder reduced the bonding strength, creating a suitable interlayer bonding agent. The proportions for the interlayer bonding material were 1:1:1.33 (epoxy resin: curing agent: putty powder), as illustrated in Fig 2B. The cohesion and internal friction angle after 28 days of solidification were 1.13 MPa and 24.6°, respectively.

### 2.2. Specimen preparation

The LIA and LT are the two factors examined in this experiment. LIA refers to the angle between the bedding plane and the horizontal plane, while LT represents the thickness in the direction perpendicular to the LIA. A full-factorial experimental method was adopted, and the design of the experimental factors is shown in Table 1.

**Table 1:** Experimental plan and results.

Sample ID	Thickness (mm)	Inclination(°)	UCS(MPa)	Peak strain(%)
**T10_D0**	10	0	8.86	0.86
**T10_D15**	10	15	7.85	3.18
**T10_D30**	10	30	7.58	5.45
**T10_D45**	10	45	7.21	6.21
**T10_D60**	10	60	0.60	2.00
**T10_D75**	10	75	8.81	1.27
**T10_D90**	10	90	12.33	1.33
**T20_D0**	20	0	9.50	0.92
**T20_D15**	20	15	7.97	1.81
**T20_D30**	20	30	7.86	2.68
**T20_D45**	20	45	5.41	2.75
**T20_D60**	20	60	0.77	1.75
**T20_D75**	20	75	7.67	1.64
**T20_D90**	20	90	11.89	1.93
**T30_D0**	30	0	10.18	1.77
**T30_D15**	30	15	7.60	1.42
**T30_D30**	30	30	7.36	2.06
**T30_D45**	30	45	5.27	2.29
**T30_D60**	30	60	0.66	0.83
**T30_D75**	30	75	6.05	1.11
**T30_D90**	30	90	13.39	1.29

The standard sample preparation procedure includes the following steps:

Step 1: Standard sample pouring. The rock-like materials were prepared according to the specified proportions and thoroughly mixed into a uniform mortar using a mixer. The mortar was slowly poured into a standard cylindrical mold with a diameter of 50 mm and height of 100 mm. After solidifying for 24 hours, the samples were removed and placed in a standard curing box at a temperature of 20 ±  2°C and a humidity of 95% for 7 days.

Step 2: Layer preparation. The specimens were fixed in a cutting machine and cut into pieces according to the parameters specified in Table 1.

Step 3: Recast specimen. The interlayer bonding material was used to bond the cut pieces of the specimens. The bonding material was evenly applied between each rock piece. To ensure consistency in the interlayer bonding strength, the amount of bonding material was controlled at 5 ±  1 g/cm².

**Fig 3:**
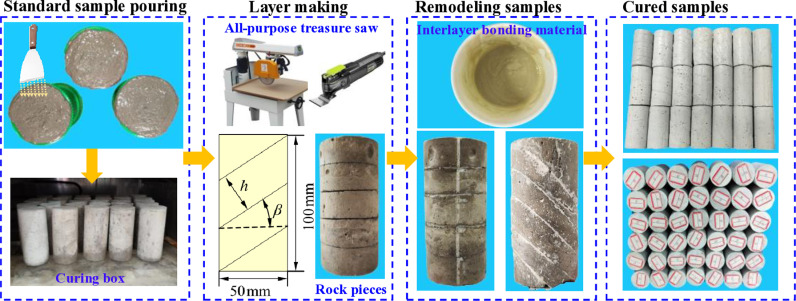
Preparation process of layered rock.

Step 4: The reconstituted specimens were placed back into the standard curing box and cured until 28d. The specific process for preparing layered rock specimens is depicted in Fig 3.

### 2.3. Test methods

**Fig 4:**
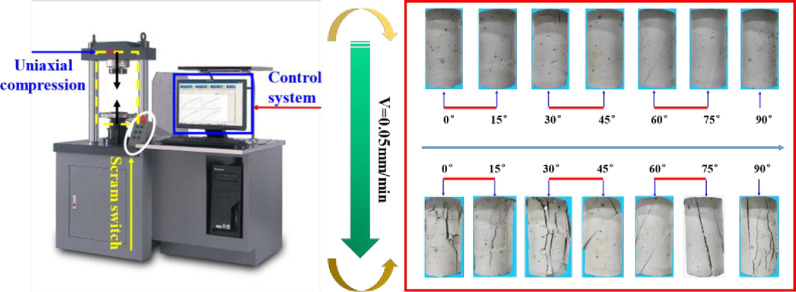
Uniaxial compression experiment process.

Uniaxial compression tests were performed on the EM EM1.305–2 testing machine, as shown in Fig 4. The loading rate was set to 0.05 mm/min, and stress-displacement data were recorded throughout the experiment.

### 2.4. Numerical modeling

**Fig 5:**
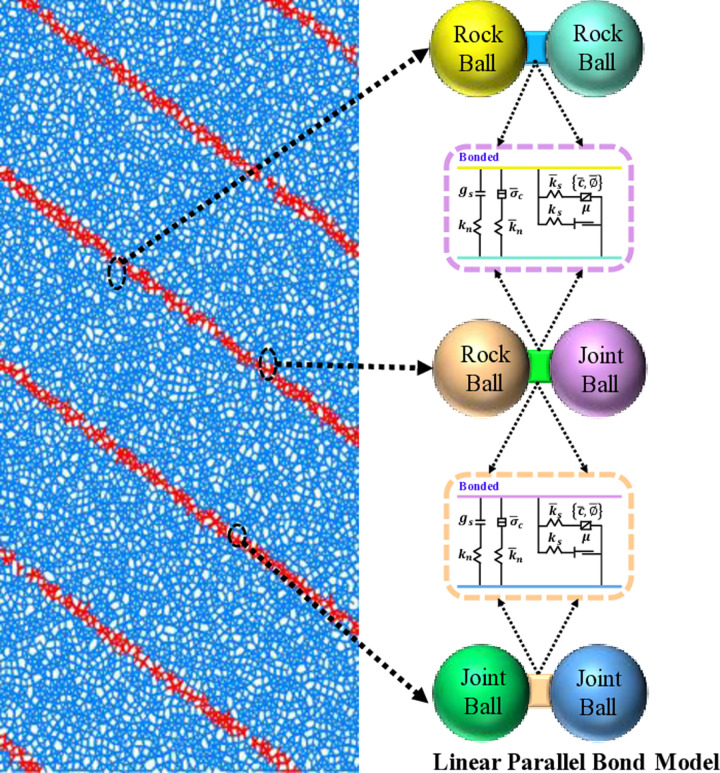
Numerical modeling of layered rocks.

Physical tests can reveal the macroscopic mechanical properties of layered rock during compression damage, but quantifying and accurately analyzing its microscopic damage is challenging. Itasca has developed PFC 2D, a discrete element numerical analysis software, specifically designed to investigate the mechanical properties and microscopic damage process of materials. The linear parallel bond model can simulate the mechanical behavior of materials during the loading process. It can consider behaviors such as opening, sliding, and rupture of bond interfaces, as well as the strength, stiffness, and deformation characteristics of the materials. Therefore, a numerical model developed using PFC2D was employed to study the microscopic damage processes. Based on the physical tests, the model categorizes the bonded particles into three types: rock to rock, rock to joint, and joint to joint, incorporating a linear parallel bond model. The numerical model with a LIA of 45° and the particle contact principles are depicted in Fig 5.

## 3. Mechanical properties

### 3.1. Stress-strain curve

**Fig 6:**
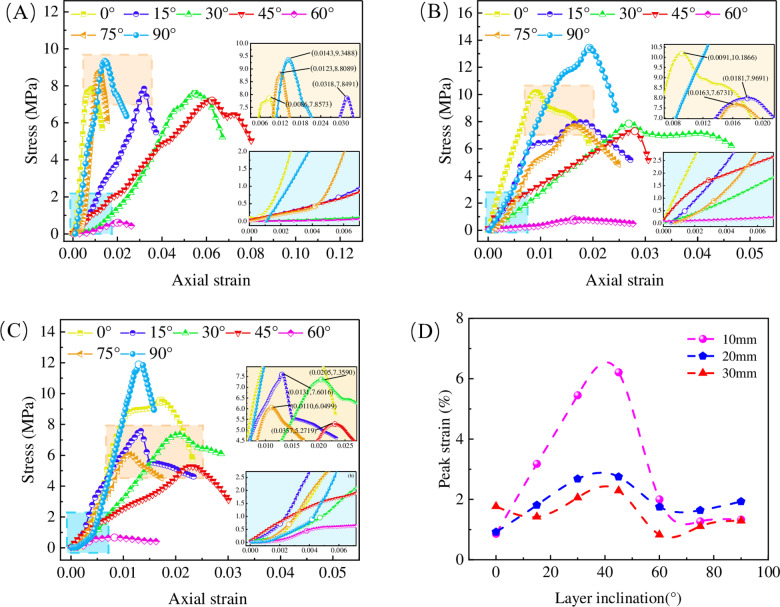
The characteristics of stress-strain curve. (A) Thickness 10 mm, (B) Thickness 20 mm, (C) Thickness 30 mm, (D) Peak strain curve.

The stress-strain curves obtained from uniaxial compression tests of layered rocks are shown in Fig 6.

As indicated in Fig 6A–C, the stress-strain curves exhibited phases of pore compaction, elastic deformation, plastic deformation, and post-peak failure, except for the specimens with a LIA of 60°.This is consistent with most rock-like materials, natural rocks, or concrete [38,39]. For the specimens with a LIA of 60°, the stress did not increase significantly with increasing strain, which is attributed to the specimen failing in the early stages of compression, with the stress during continued failure remaining low.

As shown in Fig 6D, The peak strain exhibits a wave shaped variation with increasing LIA, with the highest point occurring around 40° and the lowest point near 60°. As the LT increases, the magnitude of peak strain variation decreases. The increase in LT enhances the overall stiffness of the specimen, resulting in a more uniform stress distribution during compression, which reduces the localized strain concentration effects. Consequently, the peak strain is lowered, leading to smaller fluctuations in the curve.

### 3.2. Uniaxial compressive strength

#### 3.2.1. Effects of layer inclination.

**Fig 7:**
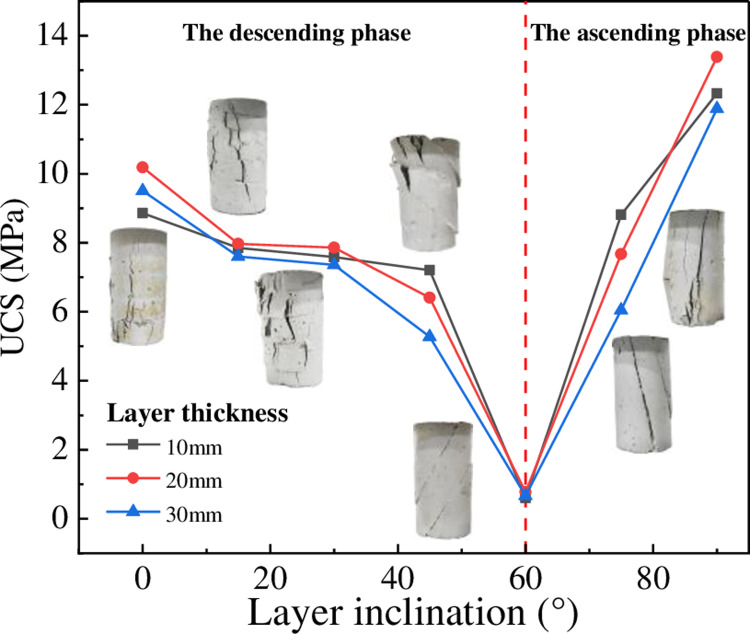
The effect of LIA on UCS.

Fig 7 shows that LIA significantly affects the uniaxial compressive strength (UCS) of layered rocks. With increase of LIA, UCS exhibits a V-shaped trend of decreasing and then increasing, the lowest point occurring at a LIA of 60°. The pattern is consistent across specimens with different LT. This is due to the variation in LIA, which results in different shear stresses on the interlayer bonding. For example, when the LIA is 0°, the interlayer bonding is subjected only to normal stress and the thickness of the bond is minimal, making it negligible during specimen deformation. However, when the LIA is π/4+φ/2 , the shear stress on the interlayer bonding reaches its maximum, and the interlayer bond strength is significantly lower than the shear strength of the rock. As a result, the interlayer bond fails first, causing the specimen to undergo sliding failure along the bedding planes.

#### 3.2.2. Effects of layer thickness.

**Fig 8:**
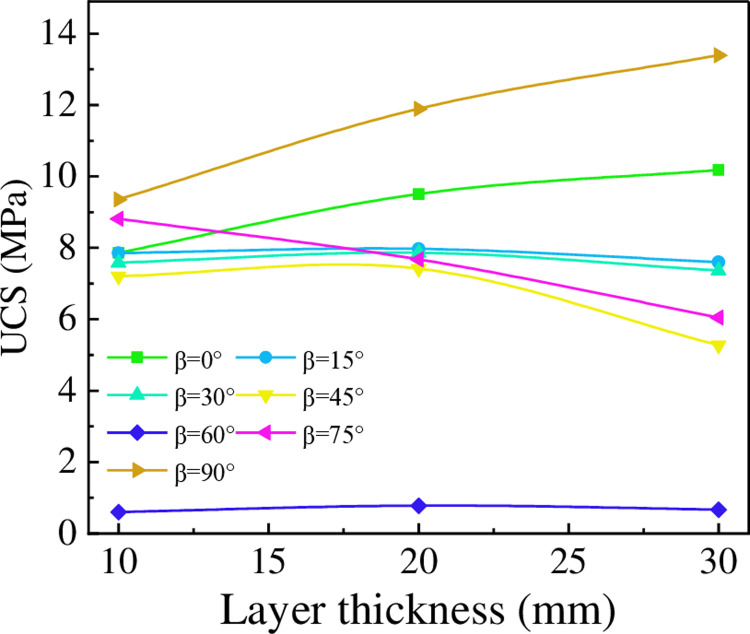
The effect of LT on UCS.

Fig 8 illustrates the variation in UCS of specimens with different LIA as the LT increases. For LIA of 0° and 90°, the UCS increases with increasing LT, as the greater thickness enhances the stiffness of the specimens, leading to more uniform stress distribution and increased resistance to deformation. For LIA of 15° and 30°, the effect of LT on UCS is not significant, as the strength gain from increasing thickness is offset by the strength reduction caused by the increasing inclination. For LIA of 45° and 75°, the UCS decreases with increasing LT. The increase in rock strength results in greater deformability, causing more deformation to be absorbed by the interlayer bonding, making the specimens more prone to failure.

### 3.3. Macroscopic failure mode

During the compression process of the specimen under external force, the initiation and propagation of cracks ultimately lead to the overall failure of the rock. In rock mechanics, crack types are typically analyzed in conjunction with failure modes.

#### 3.3.1. Macroscopic crack types.

**Fig 9:**
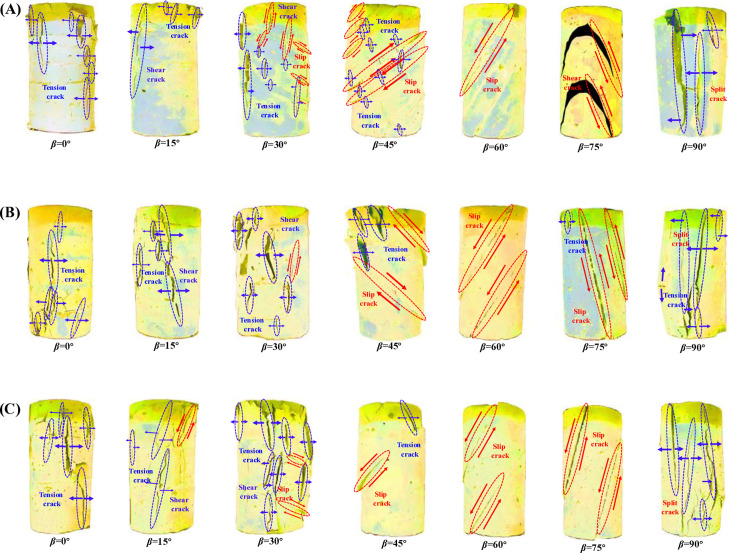
Cracks distribution and classification. (A) Thickness 10 mm, (B) Thickness 20 mm, (C) Thickness 30 mm.

The identification of crack types is based on stress analysis and provides a foundation for further classification of failure modes. After the completion of the uniaxial compression test, images of the damaged specimens were collected. Based on these images, cracks were categorized into rock-type and interlayer-type. According to the stress conditions under which the cracks formed, rock-type cracks were further divided into tensile cracks and shear cracks, while interlayer-type cracks were classified into slip cracks and split cracks. The specific distribution of these cracks is shown in Fig 9. The diagrams of these crack types are further described below:

(1) Tensile crack. When the external tensile stress exceeds the internal tensile strength of the rock, extensional cracks will develop within the rock. These cracks typically form perpendicular to the direction of the maximum tensile stress and propagate parallel to the direction of the maximum principal stress, resulting in the relative separation of the rock masses on either side. This phenomenon is most pronounced when the LIA is 0°.
(2) Shear crack. When the external shear stress exceeds the internal shear strength of the rock, shear cracks will develop within the rock. These cracks typically form along the direction of maximum shear stress and its perpendicular direction, resulting in relative displacement along the shear plane. This phenomenon can be well observed when the LIA is 15° and 30°.
(3) Slip crack. These cracks typically occur along weak planes in the rock or stratification, such as bedding planes, joints, or fault planes, resulting in relative sliding. Their characteristic feature is the relative displacement of the rock masses on either side of the crack without significant signs of failure. Cracks at a LIA of 60° are typical examples of this type of crack.
(4) Split crack. The characteristic of splitting cracks is the noticeable separation of material on either side of the crack, which typically forms perpendicular to the direction of the maximum tensile stress. This type of crack only appears in specimens with a LIA of 90°, making it a unique form of cracking.

Fig 9D shows the types of cracks generated after failure in specimens with different LIA. One or more types of cracks were present, but a single crack type typically dominates the failure. When the LIA is 0°, tensile cracks dominated failure; at 15°, both tensile and shear cracks developed, with shear cracks being dominant. When the LIA is 30° and 45°, tensile cracks, shear cracks, and slip cracks were all present, with shear cracks playing the primary role. When the LIA is 60° and 75°, only slip crack was present. While at 90°, split cracks, tensile cracks, and shear cracks occurred, and the number of crack types increases with LT. This is because the variation in LIA leads to changes in the stress distribution between the interlayer joints and the rock, resulting in different types of cracks forming.

#### 3.3.2. Macroscopic failure mode.

**Fig 10:**
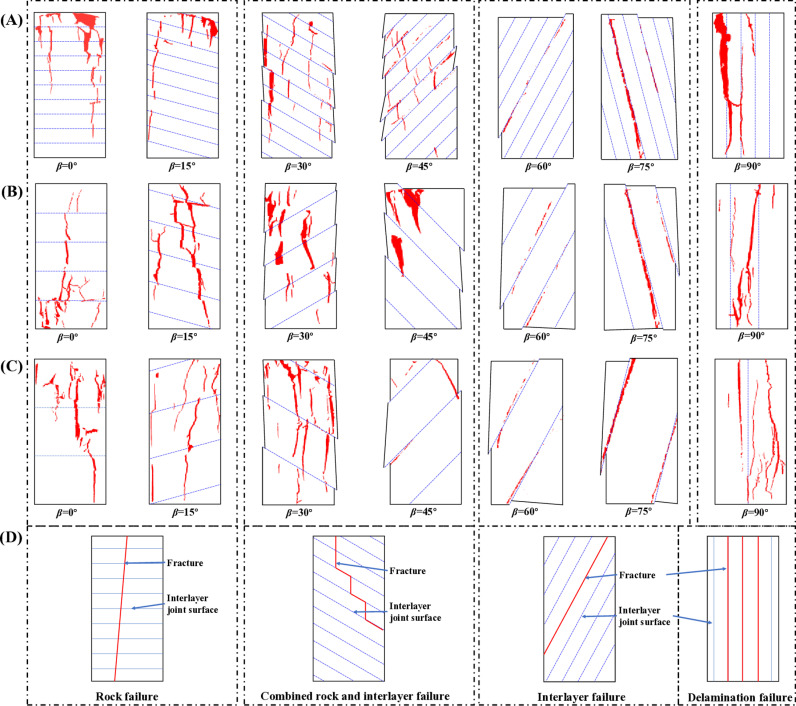
The macroscopic failure mode. (A) Thickness 10 mm, (B) Thickness 20 mm, (B) Thickness 30 mm, (D) failure modes summarized.

The generation and merging of cracks ultimately lead to the failure of the specimen, and the macroscopic crack formation constitutes the failure mode of the specimen. Based on the observed crack types in the experiments, further studies on the failure modes of the specimens were conducted. Researchers have identified more than ten failure modes [25], but some models are difficult to distinguish in practical engineering applications, which may mislead support methods or safety measures. Therefore, this study categorizes the failure modes into four types based on the primary regions where cracks occur: rock failure, combined rock and interlayer failure, interlayer failure, and delamination failure, as shown in Fig 10. The specific descriptions are as follows:

(1) Rock failure. This failure mode is caused by tensile or shear cracks in the rock portion, where the cracks can penetrate through several layers, eventually forming one or more continuous failure planes across the specimen. The rock failure model appears in specimens with LIA of 0° and 15°.
(2) Combined rock and interlayer failure. This failure mode arises from the simultaneous failure of both the rock portion and the interlayer. Due to the complex types of cracks and mechanical mechanisms involved, many sub-models have emerged. Analyzing the schematic diagrams of these sub-models, they can all be categorized under the combined rock and interlayer failure model. This is also the most commonly encountered failure model in engineering. It is notably observed in specimens with layer inclinations of 30° and 45°.
(3) Interlayer failure. It can also be referred to as the interlayer slip failure model. This failure mode is caused by slip cracks, where the rock within the specimen remains undamaged during compression, as seen in specimens with LIA of 60°and 75°. The failure modes are summarized into three types: rock failure, interlayer failure, combined rock and interlayer failure.
(4) Delamination failure. This failure mode occurs exclusively in specimens with a LIA of 90°. The interlayer tensile stress breaks the bonding, causing the specimen to separate into individual layers, which then undergo further failure through fracturing, bending, or shearing.

As the LT increases, the macroscopic failure pattern gradually becomes simpler, and the crack propagation path becomes clearer. The increase in LT results in the weakening of the anisotropic characteristics of layered rocks, causing the macroscopic failure mode to resemble that of intact rocks.

## 4. Damage mechanism

### 4.1. Numerical model validation

**Fig 11:**
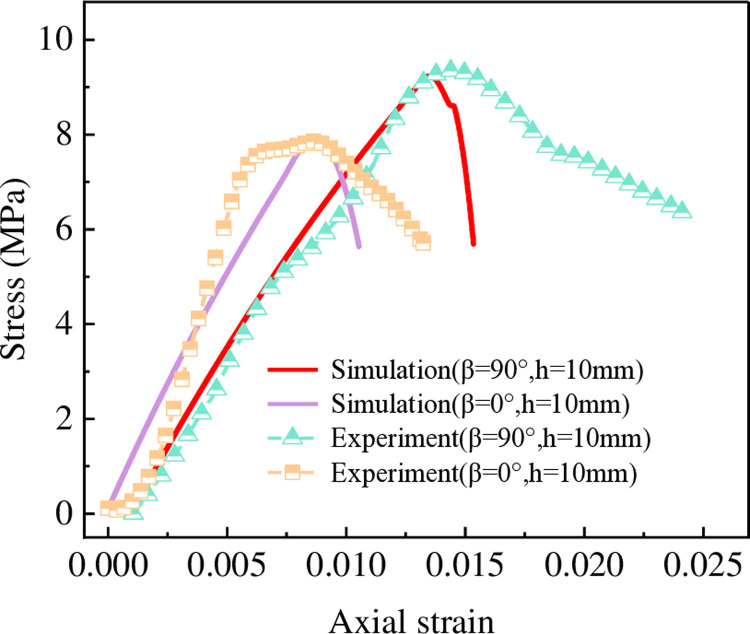
Numerical model calibration results.

A numerical model similar to physical experiments was established using PFC 2D software to study the microscopic damage of layered rock. The numerical model parameters were calibrated using a trial-and-error method until the numerical results closely matched the physical test results, as depicted in Fig 11. The specific parameters are listed in Table 2.

**Table 2:** Numerical simulation parameters.

Types	Emod/ Pa	pb_emod/ Pa	kratio	pb_ten/ Pa	pb_coh/ Pa	pb_fa/ °	fric
**Rock**	1.0 × 10^10^	1.0 × 10^10^	1.5	4.53 × 10^6^	4.53 × 10^6^	45	0.5
**Layer**	2.0 × 10^9^	2.0 × 10^9^	0.3	9.06 × 10^5^	9.06 × 10^5^	9.0	0.1
Contact	6.0 × 10^9^	6.0 × 10^9^	0.9	2.72 × 10^6^	2.72 × 10^6^	27	0.3

As illustrated in Fig 11, the stress-strain curves obtained from the numerical simulation exhibit a high degree of consistency with the experimental results. The peak strength deviations were 1.0% and 1.2%, respectively, and the peak strain deviations were within 0.05%. When the LIA is 0°and 90°, the stress-strain curve shapes are essentially consistent. Based on these findings, the model demonstrates high reliability and can accurately represent the microscopic damage mechanisms of layered rock under uniaxial compression.

### 4.2. Microscopic damage process

**Fig 12:**
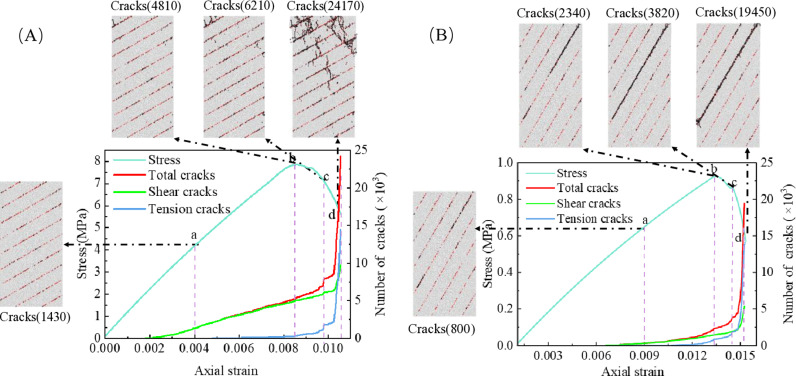
The development process of micro-cracks. (A) CRIF mode, (B) IF mode.

The gradual expansion of micro-cracks in the specimen leads to the occurrence of macroscopic failure. Preventing the rapid propagation of micro-cracks is crucial for ensuring macroscopic stability. Taking LIA of 30° and 60° as examples, the development of micro-cracks under CRIF mode and IF mode is investigated, as shown in Fig 12.

As depicted in Fig 12, the development of internal micro-cracks within the specimen can be categorized into three distinct stages: no crack formation, crack slow growth, and crack rapid propagation. During the no-crack stage, the external load remains below the bonding strength between particles, thereby preventing any adhesion failure between them and ensuring that no cracks are formed. As the load increases, the number and location of crack formation are significantly influenced by the LIA. Fig 12A illustrates the crack propagation process in CRIF mode. At point a, cracks are distributed along the interlayers. At point b, micro-cracks begin to appear in the rock portion. At point c, the number of micro-cracks increases mainly in the rock portion. At point d, micro-cracks in the rock portion penetrate to form macroscopic fractures. Fig 12B represents the crack propagation process in IF mode, where it can be observed that as the load increases, micro-cracks develop exclusively along the interlayers, with no cracks forming in the rock portion. The failure in this mode is characterized by the overall sliding of the rock layers after the interlayer bonding fractures. Changes in LIA alter the stress distribution on weak interlayer planes, limiting its deformation and maximizing the rock’s load-bearing capacity, thereby enhancing its overall strength. Therefore, for the support strategy of layered rock, reinforcement should be applied promptly in the early stage of interlayer bonding fracture.

### 4.3. Crack distribution characteristics

**Fig 13:**
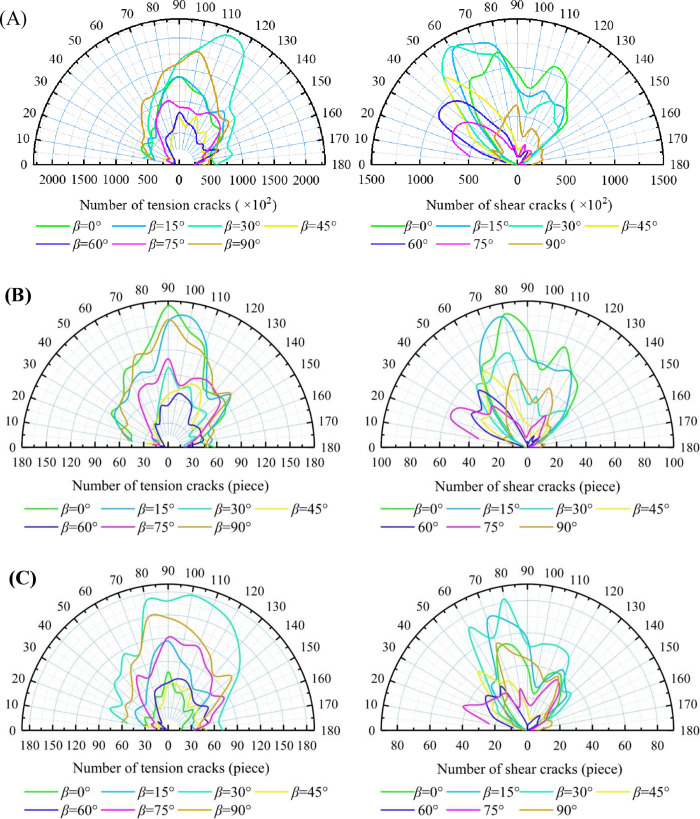
The distribution characteristics of micro-cracks. (A) Thickness 10 mm, (B) Thickness 20 mm, (C) Thickness 30 mm.

Statistical analysis of micro-crack quantity and orientation offers a deeper understanding of the microscopic damage characteristics of layered rock with varying LIA, providing a reliable basis for assessing macroscopic failure modes. Fig 13 shows the relationship between crack inclination and quantity, with tensile cracks on the left and shear cracks on the right.

The distribution of tensile cracks exhibits a clear concentration, with the highest density occurring between 70° and 110°. This is particularly evident in specimens with a 20 mm LT, where the dominant crack orientation is around 90°. The influence of LT on crack inclination distribution is minimal, as tensile cracks primarily form in the rock portion. During compression, the propagation of these cracks aligns with the principal stress direction, appearing as extension along the 90° axis in a two-dimensional plane.

The distribution of shear cracks indicates that their orientation is more dependent on the LIA. At LIA of 0°, 15°, and 90°, micro-cracks exhibit a bimodal distribution, concentrated around 60° to 80° and near 110°. This occurs because shear failure primarily takes place in the rock portion, where the lower strength of slate leads to simultaneous shear failure on both sides. When the LIA is 30° and 45°, the micro-cracks show a significantly dispersed distribution, as shear behavior occurs both within the rock and along the layer interfaces, without a dominant failure mechanism. When the LIA increases to 30°, 45°, 60° and 75°, the shear cracks are concentrated at the layer surfaces, indicating that shear cracks tend to develop along the interlayer surfaces. The distribution of shear cracks correlates well with the failure modes, further confirming the validity of the four failure mode classifications.

### 4.4. Crack propagation mechanism

**Fig 14:**
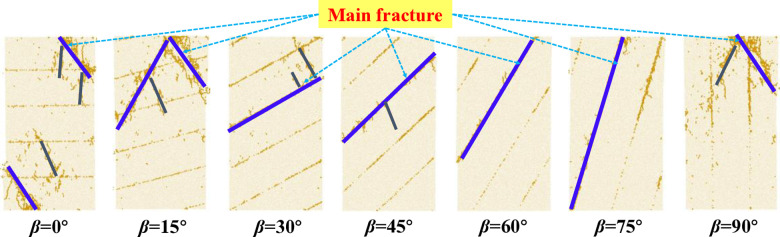
The effect of layer inclinations on the distribution of main fracture.

Fig 14 illustrates the crack distribution following specimens damage with varying LIA. The main crack penetrates the specimen, which is the primary cause of failure.

As shown in Fig 14, for specimens with LIA of 0°, 15°, and 90°, cracks mainly propagate along one or two layer surfaces within the rock, eventually forming a major crack that penetrates the interlayer joints. In specimens with LIA of 30° and 45°, Although failure of the rock portion and the interlayer occurs simultaneously, the main cracks is interlayer failure. The secondary cracks form within the rock portion, which do not penetrate the entire specimen, mainly accommodating compressive deformation. In specimens with LIA of 60° and 75°, the main cracks are also along the interlayer joints, with no significant micro-cracks in the rock portion. Therefore, interlayer sliding remains the primary cause of failure. As the strength of the rock portion increases, interlayer sliding failure will become more pronounced. Therefore, when designing underground engineering projects in layered rock, priority should be given to the relationship between the LIA and the project layout.

## 5. Conclusions

In order to investigate the effect of layered structure on the mechanical properties of layered rock. This paper is devoted to the first uniaxial compression test using mountain sand, cement and gypsum to prepare layered rock specimens. At the same time, the effects of LIA and LT on the micro-damage mechanism of layered rock were investigated by using PFC 2D numerical simulation. The main conclusions are as follows.

(1) The stress-strain variation pattern of specimens prepared with rock-like materials is consistent with that of typical rocks. The rock reconstruction method is suitable for experimental studies of layered rock specimens.
(2) LIA significantly affects the strength of the specimen. As the LIA increases, the UCS shows an approximately V-shaped trend, with the lowest point occurring at 60°. The effect of LT on UCS is not significant, as the strength gain from increasing thickness is offset by the strength reduction caused by the increasing inclination.
(3) Through image analysis of the damaged specimens, four crack types and four failure modes can be identified and are highly correlated with the LIA. With the gradual increase of LIA, the layered rock failure initiated by tension cracks gradually shifted to shear cracks leading to rock and layered failure, sliding cracks leading to layered failure and separation cracks leading to layered failure.
(4) Numerical simulation results indicate that cracks first form along the interlayer joints in samples with different Layered characteristics. The direction of tensile crack propagation aligns with the loading direction. Shear cracks are significantly influenced by the relationship between the LIA and the loading direction. For LIA of 0°, 15°, and 90°, rock failure is the primary factor in the damage of the samples. When LIA is greater than 15°and less than 90°, the main cracks are generated by interlayer bonding failure, accompanied by tensile or shear failure of the rock.

In summary, the paper provides an in-depth examination of the failure behavior of layered rocks under uniaxial compression. The results from physical tests and numerical simulations reveal the mechanisms of crack extension and damage, providing a foundation for preliminary design and support measures in layered rock engineering.
